# Dopamine signaling impairs ROS modulation by mitochondrial hexokinase in human neural progenitor cells

**DOI:** 10.1042/BSR20211191

**Published:** 2021-12-08

**Authors:** Gabriela Assis-de-Lemos, Jamila Monteiro, Viviane M. Oliveira-Valença, Guilherme A. Melo, Ricardo A. de Melo Reis, Stevens K. Rehen, Mariana S. Silveira, Antonio Galina

**Affiliations:** 1 Laboratory of Bioenergetics and Mitochondrial Physiology, Institute of Medical Biochemistry Leopoldo de Meis, Center for Health Sciences, Federal University of Rio de Janeiro (UFRJ), Brazil; 2 Laboratory of Neurogenesis, Institute of Biophysics Carlos Chagas Filho, Center for Health Sciences, Federal University of Rio de Janeiro (UFRJ), Brazil; 3 Laboratory of Immunology, Institute of Biophysics Carlos Chagas Filho, Center for Health Sciences, Federal University of Rio de Janeiro (UFRJ), Brazil; 4 Laboratory of Neurochemistry, Institute of Biophysics Carlos Chagas Filho, Center for Health Sciences, Federal University of Rio de Janeiro (UFRJ), Brazil; 5 D'Or Institute for Research and Education (IDOR), Brazil; Institute of Biomedical Sciences, Center for Health Sciences, Federal University of Rio de Janeiro (UFRJ), Brazil

**Keywords:** dopamine, mitochondria, mt-HK, neurodevelopment, ROS

## Abstract

Dopamine signaling has numerous roles during brain development. In addition, alterations in dopamine signaling may be also involved in the pathophysiology of psychiatric disorders. Neurodevelopment is modulated in multiple steps by reactive oxygen species (ROS), byproducts of oxidative metabolism that are signaling factors involved in proliferation, differentiation, and migration. Hexokinase (HK), when associated with the mitochondria (mt-HK), is a potent modulator of the generation of mitochondrial ROS in the brain. In the present study, we investigated whether dopamine could affect both the activity and redox function of mt-HK in human neural progenitor cells (NPCs). We found that dopamine signaling via D_1_R decreases mt-HK activity and impairs ROS modulation, which is followed by an expressive release of H_2_O_2_ and impairment in calcium handling by the mitochondria. Nevertheless, mitochondrial respiration is not affected, suggesting specificity for dopamine on mt-HK function. In neural stem cells (NSCs) derived from induced-pluripotent stem cells (iPSCs) of schizophrenia patients, mt-HK is unable to decrease mitochondrial ROS, in contrast with NSCs derived from healthy individuals. Our data point to mitochondrial hexokinase as a novel target of dopaminergic signaling, as well as a redox modulator in human neural progenitor cells, which may be relevant to the pathophysiology of neurodevelopmental disorders such as schizophrenia.

## Introduction

Dopamine (DA) is an important neurotransmitter in multiple areas of the adult brain. DA acts through specific G-protein–coupled receptors, which are divided into D1- and D2-like families and mediate both excitatory and inhibitory neurotransmission, mainly via the modulation of the cAMP pathway [1]. Dopamine has also been studied from a neurodevelopmental perspective because DA receptors are expressed very early in the development of the central nervous system before synaptogenesis [2,3]. Currently, dopaminergic signaling is known to modulate crucial processes, such as the regulation of gene expression, proliferation, and differentiation [4]. Altered dopamine signaling during ontogenesis may be involved in the aetiology of neuropsychiatric disorders [5]. Expression of elevated levels of D2 dopaminergic receptors during development in transgenic mice alters dopamine signaling that leads to abnormal behavior and impaired working memory even when receptor density is normalized in the adult animals [6]. Similarly, a transient increase in dopaminergic signaling during a critical window in neurodevelopment of *Drosophila melanogaster* increased visual responsiveness and locomotion in adult animals and reduced gamma-like activity, resembling the symptoms observed in patients with schizophrenia [7,8]. In contrast, a transient increase in dopaminergic activity in adult animals had no effect on visual responsiveness, indicating strict developmental nature of these effects.

Reactive oxygen species (ROS), such as O^•−^ and H_2_O_2_, are natural byproducts of oxidative metabolism, which can modulate the development of the brain [9,10]. ROS are traditionally correlated with oxidative damage and cell death in physiological and pathological contexts, such as cancer, neurodegenerative and psychiatric disorders. Oxidative stress has been implicated both with symptomatic features [11,12] and in etiology of psychiatric disorders [13]. However, ROS are recognized as important intracellular signals that regulate many signaling pathways at physiological levels by modulation of redox-sensitive molecules, such as transcription factors, signaling proteins and cytoskeletal components [14]. ROS signaling during neurodevelopment has been shown to modulate the proliferation, migration, and differentiation of neural progenitor cells [15,16]. Intracellular redox balance is dynamically regulated by modulation of ROS production and scavenging systems. Moreover, cytosolic levels of ROS may be controlled by ROS release through the plasma membrane into the extracellular medium. Extracellular ROS may participate in intercellular communication, leading to the activation of redox-sensitive target proteins. Interestingly, ROS modulate signaling pathways and cell function in dopaminergic neurons, where both exogenous and endogenous H_2_O_2_ reversibly suppress dopamine release [17,18].

Mitochondria are the main source of cellular ROS [19,20], which may be produced at various sites of the electron transport system (ETS) or by matrix dehydrogenases [21]. Mitochondria have their own pool of antioxidant enzymes, such as superoxide dismutase, catalase, glutathione and thioredoxin systems; however, brain mitochondria are characterized by high rates of ROS production compared with other tissues [22]. Glucose is the main energy substrate in the brain and may be a significant source of ROS under pathological conditions [23]. More than 90% of glucose in the central nervous system is phosphorylated by hexokinase (HK) at the mitochondrial surface. HK bound to the mitochondria acquires high preference for ATP generated by oxidative phosphorylation (OxPhos) and functionally couples glucose and oxygen metabolism [24].

In 2004, our group demonstrated that HK bound to the outer mitochondrial membrane (OMM) in cultured neurons and brain is a potent regulator of ROS produced by ETS [25]. The reaction catalyzed by HK generates ADP, which is a substrate for ATP production, and thus consumes mitochondrial membrane potential (mΔψ) [25]. Generation of mitochondrial ROS by ETS is highly dependent on mΔψ [26]; thus, the activity of mitochondria-bound hexokinase (mt-HK) almost completely abolishes intraneuronal ROS levels in high glucose medium [25], indicating that mt-HK is a powerful modulator of ROS in the central nervous system.

The control of the production of ROS may determine brain development because imbalance in mitochondrial ROS due to a mutation in SOD2 (mitochondrial isoform of the antioxidant enzyme superoxide dismutase) leads to abnormal brain morphology [27]. Therefore, mitochondrial ROS and their regulation by mt-HK are important for neurodevelopment [28]. Our group showed that mt-HK activity linearly increases throughout neurodevelopmental stages [29], correlating with oxidative metabolism and mitochondrial ROS production, unlike classical antioxidant systems [30]. This observation suggests that mt-HK may also modulate mitochondrial ROS during brain development.

Dopaminergic signaling in the context of neurodevelopment is poorly understood, especially with regards to cellular redox dynamics. Thus, our goal was to investigate whether dopamine modulates the redox function of mt-HK in human neural progenitor cells (NPCs). Our data suggest that mt-HK is a downstream target of dopaminergic signaling in NPCs. Dopamine decreases mt-HK activity, which impact redox balance in these cells. Finally, we propose that modulation of mt-HK function might be involved in the pathophysiology of brain disorders.

## Materials and methods

### Materials

Amplex Red (Cat #A22177), MitoSOX Red (Cat #M36008) and Alexa Fluor 488 secondary antibody (Cat #A11008, RRID:AB_143165) were purchased from Molecular Probes (Thermofisher). N2 supplement (Cat #17502001), B27 supplement (Cat #17504044), Penicillin-Streptomycin (Cat #15140122), Laminin (Cat #23017015), Neural Induction supplement, StemPro Accutase (Cat #A11105), Geltrex (Cat #A1413301), and Calcium Green-5N (Cat #C3737) were from Gibco (Thermofisher). Dihydroethidium and Alexa Fluor 594 secondary antibody (Cat #A11032, RRID:AB_141672) were from Molecular Probes. D_1_R antibody (Cat #324390) was from Millipore; D_2_R antibody (Cat # sc-5303, RRID:AB_668816) was from Santa Cruz Biotechnology; Poly-l-ornithine (Cat #P4957), Dopamine (Cat #H8502), SCH 23390 (Cat #D054), Raclopride (Cat #R121), Protease Inhibitor Cocktail (Cat #P8340) and all other reagents were from Sigma-Aldrich.

### Cell cultures

Human neural progenitor cells (NPCs) were differentiated from the embryonic stem cell (ESC) line BR1 [31] following the protocol described by Chambers and collaborators [32]. Briefly, BR1 cells were dissociated into single cells and replated onto Matrigel-coated dishes. After 72 h, cells were switched from ESCs conditions to knockout serum replacement medium containing inhibitors of SMAD signaling (Noggin, SB431542 or both factors) and allowed to differentiate into NPCs for a total of 11 days. NPCs were cultured in DMEM/F-12 (Dulbecco’s modified Eagle’s Medium/Nutrient Mixture F12, (Thermofisher/Gibco) supplemented with 2% N2 supplement, 1% B27 supplement, 1% penicillin-streptomycin (10,000 U/ml) (NPC medium) in plates pretreated with Poly-l-ornithine and Laminin. The cells were maintained at 37°C in 95% air–5% CO_2_ in a humidified incubator. Experiments were performed when the plates achieved around 80% confluence. The maximum number of cell passages used was 16.

We also performed experiments in neural stem cells (NSCs) derived from induced-pluripotent stem cells (iPSCs) from fibroblast biopsies of three schizophrenia patients (EZQ4, GM23760B and GM23761B cell lines) versus three healthy individuals (CF1, CF2 and GM23279A cell lines). Lines GM23279A, GM23760B and GM23761B were bought from Coriell Institute for Medical Research (New Jersey, U.S.A.). Lines CF1, CF2 and EZQ4 were derived from skin biopsies collected at D’Or Institute for Research and Education (IDOR) by Dr Mário André da Cunha Saporta, specialist in biopsy procedures, with informed consent obtained from the patients and/or their legal tutors. All procedures for sample collection were conducted in accordance with the World Medical Association Declaration of Helsinki and were approved by the IDOR Ethics Committee under the number CAAE 32385314.9.0000.5249. The collected cells were used to generate iPSCs from which the NSCs CF1, CF2 and EZQ4 were specified.

NSCs were cultured at 49% Advanced DMEM/F12 (Gibco) + 49% Neurobasal Medium (Gibco) supplemented with 2% Neural Induction Supplement (NEM medium) in plates pretreated with Geltrex. iPSCs lines were differentiated into NSCs according to Neural Induction Gibco’s protocol [33].

### Cell treatment

Since using antioxidant in the dilution of dopamine would interfere in the levels of cellular ROS, to minimize its autooxidation dopamine was maintained in the dark and diluted in NPC medium just before treating the NPCs. Nevertheless, measuring dopamine autofluorescence (excitation wavelength of 265 nm/ emission wavelength at 350 nm), we observed oxidation of ∼45% of dopamine after 48 h (data not shown). The ‘control NPCs’ group includes NPCs exposed only to the vehicle of dopamine dilution (NPC medium).

The protocol used for the treatment of NPCs with dopamine was based on a previous study, which investigated the effect of dopamine treatment on mitochondrial function of the human neuroblastoma cell line SH-SY5Y [34]. The NPCs were treated with 100 µM dopamine and maintained for 48 h at 37°C. Then, the medium was discarded, cells were washed with DMEM/F12 and the biochemical analyses were performed, in comparison with control NPCs. For the experiments using the antagonists of dopamine receptors, NPCs were pretreated for 10–15 min with specific antagonist for D_1_R, SCH 23390 (10 µM) or D_2_R, Raclopride (20 µM) and then treated with dopamine for 48 h.

### High resolution respirometry

Oxygen consumption assays were conducted using High Resolution Respirometry (Oroboros O2K, Innsbruck, Austria), at 37°C with constant stirring. Cells were enzymatically detached from the plate (StemPro Accutase) and used at 1 × 10^6^ cells/ml in NPC medium. Modulators of mitochondrial function were added sequentially, always after signal stabilization, as follows: Oligomycin 1 µg/ml, FCCP in 1 µM titration, Antimycin A 1 µM. Data were collected through a specific software that shows, in real time, the concentration of oxygen and the specific oxygen flow, which means the negative time derivative of oxygen concentration (DatLab software 5.0, Oroboros Instruments, Innsbruck, Austria).

### Intracellular O^•−^ production

Intracellular O^•−^ production of the NPCs was accessed by DHE (10 µM) that once oxidized by O^•−^ intercalates with the DNA, staining cell nucleus, and emits at 605 nm. Briefly, cells were plated in a 96w µClear plate, where the dopamine treatment and the experiment were performed. NPCs were incubated with DHE for 40 min at 37°C in the incubator, after which the dye was washed out before imaging. To evaluate mt-HK capacity to modulate ROS, the cells were coincubated with 2-deoxyglucose (2-DOG) 35 mM, an activator of hexokinase activity, and ATP 5 mM during the incubation time with DHE, and compared with cells incubated with vehicle of 2-DOG dilution (distilled water). 2-DOG is a substrate of hexokinase that is phosphorylated but, instead of G6P, its reaction product is 2-deoxyglucose-6-P, which does not inhibit hexokinase activity in the same range as G6P [35,36]. The inclusion of 2-DOG is a strategy to specifically evaluate the impact of glucose phosphorylation in mitochondria by mt-HK on mitochondrial ROS production [25,29,37,38]. The images were obtained with Operetta (Perkin Elmer) using Harmony software, at 37°C and 5% CO_2_.

### Extracellular H_2_O_2_ production

The H_2_O_2_ release to extracellular space was measured with Amplex Red (AmR) probe using a fluorimeter (Varian Cary Eclipse; Agilent Technologies, Santa Clara, CA). Fluorescence was detected at an excitation wavelength of 563 nm (slit 5 nm) and an emission wavelength of 587 nm in NPC medium in the presence of 2.5 × 10^6^ cells/ml and 2 U/ml horseradish peroxidase. Once AmR is cell membrane impermeant, only extracellular H_2_O_2_ was measured with this method. To transform fluorescence units into specific H_2_O_2_ production, we used a calibration curve with known amounts of H_2_O_2_. To access maximal H_2_O_2_ production, all experiments were done in the presence of SOD 30 U/ml.

### Immunofluorescence

Briefly, the NPCs were cultured and treated in a 96w µClear plate, where they were fixed with 4% paraformaldehyde, permeabilized with Triton X-100 0.3% and incubated overnight with anti-D_1_R (1:100) and anti-D_2_R (1:800) at 4°C. After incubation with primary antibodies, the cells were incubated for 1 h with Alexa Fluor 488 and Alexa Fluor 594 secondary antibodies (1:400). The nucleus staining was made with 1 μg/ml of 4′.6′-diamino-2-phenylindole (DAPI). The negative control was obtained after incubation with the secondary antibodies in the absence of the primary antibodies. Images were obtained in Operetta (Perkin Elmer) using Harmony software.

### Total hexokinase activity

Total HK activity was performed by the reduction of NAD^+^ measured fluorimetrically. Fluorescence was detected at an excitation wavelength of 352 nm (slit 10 nm) and an emission wavelength of 464 nm in the coupled reaction with exogenous G6PDH from L. *mesenteroides*, as described previously [39]. The measurement was done using a Varian Cary Eclipse fluorimeter. To assess HK activity, the cells (around 3 × 10^6^ cells) were permeabilized in the presence of 2 mM pyruvate, 2 mM malate and 10 mM glutamate with 0.002% digitonin. Then, the cells were centrifuged at 4°C, resuspended in the assay medium and kept on ice. The assay medium contained 10 mM Tris-HCl pH 7.4, 320 mM mannitol, 24 mM MgCl_2_, 0.08 mM EDTA, 1 mM EGTA, 8 mM Pi, 1 mM ATP, 10 µM AP5A, 1 U/ml G6PDH, 1 mM β-NAD+ and the reaction started upon the addition of 5 mM glucose.

### Mitochondrial hexokinase activity coupled to the oxidative phosphorylation

Mt-HK activity was measured using a Perkin-Elmer Victor spectrophotometer microplate reader, in a 96w plate, with detection of NADH at 340 nm. The cells were permeabilized in the same protocol as described for total HK activity and used in the approximate concentration of 1 × 10^6^ cells/well. The assay medium contained 10 mM Tris-HCl pH 7.4, 320 mM mannitol, 24 mM MgCl_2_, 0.08 mM EDTA, 1 mM EGTA, 8 mM Pi, 1 mM glucose, 0.3 mM ADP, 10 µM AP5A, 1 U/ml G6PDH, 1 mM β-NAD+ and the reaction started with the addition of 5 mM succinate. As a control of OxPhos-coupled mt-HK activity, we used 1 µg/ml oligomycin, an ATP-synthase inhibitor which is expected to abolish mt-HK activity due to the lack of mitochondrial ATP.

### Calcium uptake assays

To measure mitochondrial calcium handling we used the calcium sensitive probe Calcium Green 5N at 200 nM with excitation/emission wavelengths of 505/535 nm using a Varian Cary Eclipse fluorimeter at 37°C. Cells were permeabilized with digitonin as described for HK activity assay. NPCs were used at 10 × 10^6^ cells/ml in a 1 ml cuvette in MIR05 without EGTA and with 10 mM Succinate, 10 µM Ap5A, 100 µM ADP and 200 µM ATP. Along the experiment, each CaCl_2_ addition resulted on a fluorescence peak representing the binding of the ion to the fluorescent probe (Figure 6A). Over time, a reduction in fluorescence is observed, indicating calcium detachment from the probe and its entry into the mitochondria. Titrations were performed until there was no more mitochondrial calcium uptake. To investigate the role of mt-HK activity in calcium uptake of control and dopamine-treated NPCs, we used a 2-DOG free condition versus the presence of 2-DOG 35 mM during the experiment.

### Flow cytometry

To access mitochondrial O^•−^ production in NSCs derived from healthy and schizophrenic individuals we used MitoSOX Red (2.5 µM), which is oxidized by O^•−^ and binds to nucleic acids, emitting its maximal fluorescence at 580 nm. Briefly, after about 3 days in culture the NSCs were enzymatically harvested of the well (six-well plate) and co-incubated for 20 min in the bath at 37°C with MitoSOX Red and 35 mM 2-DOG or Antimycin A, as a positive control of mitochondrial O^•−^ production. The inclusion of 2-DOG is a strategy to specifically evaluate the impact of glucose phosphorylation in mitochondria by mt-HK on mitochondrial ROS production [25,29,37,38]. After the incubation time, the cells were washed twice with warm PBS and analyzed in a BD FACSCalibur Flow Cytometer. The control for the intrinsic fluorescence background of the cells was obtained including a group exposed only to the vehicle of MitoSOX Red dilution (NPC medium).

### Statistics

Data were plotted on GraphPad Prism 6® or OriginLab 8 software, and are expressed in mean ± SEM. The *n* number indicates the number of independent cell culture preparations for NPCs and the number of individuals for NSCs (three control versus three schizophrenia patients). Representative experiments are shown for a sake of clarity of the data. All data were quantified from at least three experiments.

Most of the data represents a comparison between two groups: control and dopamine-treated NPCs, and the statistical analysis used was unpaired *t*-test. The data comparing NSCs derived from healthy versus schizophrenic individuals were also analyzed using unpaired *t*-test. The data with unequal variances were analyzed using unpaired *t*-test with Welches correction. The data from DRs antagonists were analyzed by one-way ANOVA with Tukey’s as a post-test for multiple comparisons. To reach statistical power, a minimum number of three experiments was necessary.

## Results

### NPCs exposed to dopamine release high levels of H_2_O_2_ without changes in intracellular ROS

**Figure 1:**
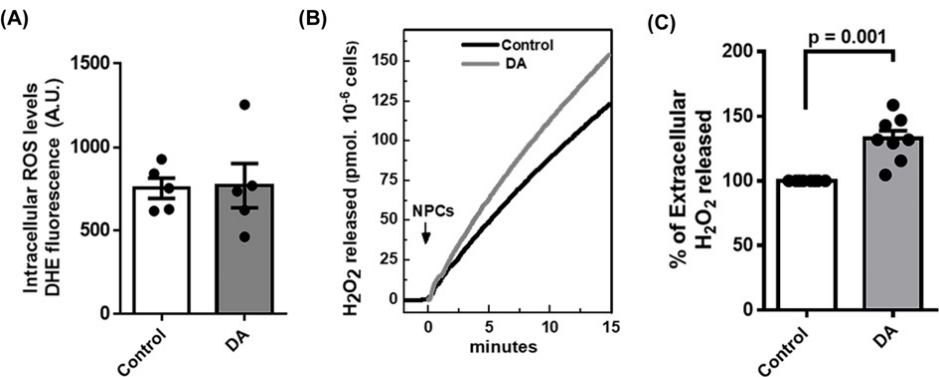
NPCs release higher levels of H_2_O_2_ upon dopamine treatment but no difference is detected in intracellular O^●−^ (**A**) Quantification of the intracellular levels of O^●-^ by DHE fluorescence of control and dopamine-treated NPCs (Dopamine 100 µM for 48 h). (**B**) Representative measurement of H_2_O_2_ release rate assessed fluorimetrically by Amplex Red-Peroxidase system, specific for H_2_O_2_, in intact NPCs. (**C**) Quantification of the rate of H_2_O_2_ release upon dopamine treatment when compared to control. In all experimental conditions we used 5 × 10^4^ cells/ml. The difference between groups was analyzed by unpaired *t*-test in a (*n* = 5 per group) and c (*n* = 7 per group). In c, we used Welches correction for unequal variances.

Previous studies have shown that dopamine may induce an increase in ROS and oxidative stress in neural cells [40,41]. We evaluated the intracellular content and release of ROS to extracellular space in control and dopamine-treated NPCs. Determination of intracellular O^•−^ using dihydroethidium (DHE), a permeable probe oxidized into a fluorescent adduct, indicated the lack of differences between control and dopamine-treated NPCs (Figure 1A). Interestingly, despite similar levels of intracellular ROS, NPCs exposed to dopamine released a higher level of H_2_O_2_ than controls (Figure 1B,C).

### Dopamine treatment impairs ROS modulation by mitochondrial hexokinase

**Figure 2:**
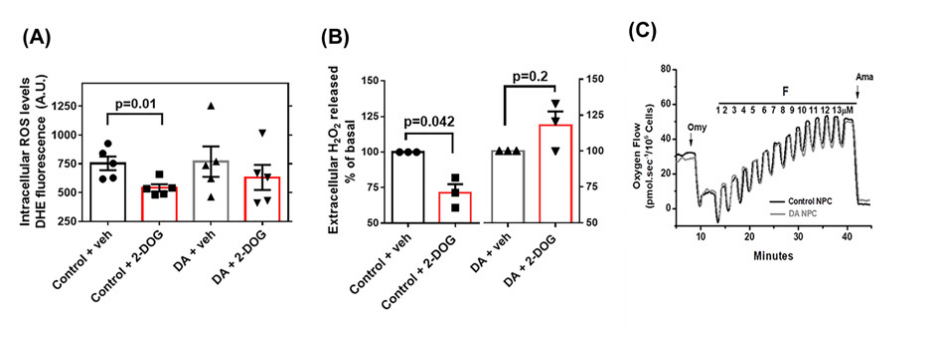
Dopamine treatment impairs the regulation of ROS by mitochondrial hexokinase with no change in mitochondrial function (**A**) Quantification of the intracellular levels of O^●−^ by DHE fluorescence after activation of hexokinase with 2-DOG 35 mM for 30 min in control versus dopamine-treated NPCs. In all experimental conditions we used 5 × 10^4^ cells/ml. (**B**) Normalized quantification of the rate of H_2_O_2_ release after the activation of hexokinase with 2-DOG 35 mM for 30 min in control versus dopamine-treated NPCs. For a sake of clarity, the scale range chosen was from 50 to 160. The differences between groups were analyzed by unpaired *t*-test in a (*n* = 5 per group) and by unpaired *t*-test with Welches correction in b (*n* = 3 per group). (**C**) Representative oxygraphic data of mitochondrial function from control and dopamine-treated NPCs in response to sequential additions of 1 µg/ml Oligomycin (Omy), 1 µM pulses of FCCP (F) and 2.5 µM antimycin A (Ama); Veh, vehicle.

Mitochondrial HK phosphorylates glucose to potently decrease ROS generation in the brain [25]; thus, we assessed ROS generation in response to the activation of mt-HK to investigate whether higher H_2_O_2_ release is influenced by mt-HK activity. Similar to the results obtained in mature neurons [25], activation of mt-HK in control NPCs by 2-DOG prevented the generation of ROS by mitochondria because significantly lower levels of ROS were detected in the intracellular and extracellular media (Figure 2A,B). Interestingly, this effect was lost in dopamine-treated NPCs, in which 2-DOG-induced activation of mt-HK did not alter intra- or extracellular ROS levels (Figure 2A,B). High-resolution respirometry of control and dopamine-treated NPCs was used to determine whether this effect is related to alterations in mitochondrial function. All tested parameters of mitochondrial function were similar (Figure 2C, representative data), suggesting that dopamine specifically acts on mt-HK to control ROS production.

### Dopamine treatment reduces the enzymatic activity of mitochondrial hexokinase in NPCs

**Figure 3:**
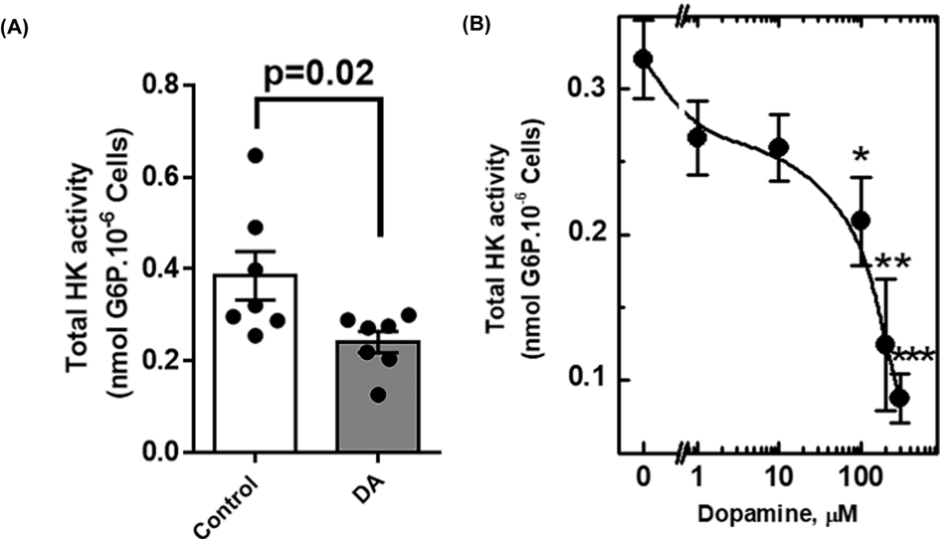
Dopamine reduces total hexokinase activity in NPCs (**A**) Total hexokinase activity of control and dopamine-treated NPCs, measured fluorimetrically through the detection of glucose-6-phosphate (G6P). (**B**) Dose–response curve of the effect of dopamine in total HK activity in NPCs (48 h). The asterisks mark the points where the inhibition of the total activity of HK is statistically significant, as * means *P*<0.05; ** means* P*<0.002; *** means *P*<0.003. The difference between groups was analyzed by unpaired *t*-test.

Since mt-HK does not decrease mitochondrial ROS in dopamine-treated NPCs, we determined specifically the mt-HK activity. Total hexokinase activity in the presence of glucose and ATP (not linked to OxPhos activity) was approximately two-fold lower in dopamine-treated NPCs than that in control NPCs (Figure 3A). A dose-response curve was used to characterize the effect of dopamine on hexokinase activity; the results indicated dose-dependent inhibition with a maximal effect at 500 µM (Figure 3B). Sigmoidal shape of the inhibition curve of HK activity versus dopamine concentration suggested that this effect is mediated by receptor activation [42].

### The inhibition of coupled activity of mt-HK by dopamine depends on the activation of D1 receptor

**Figure 4:**
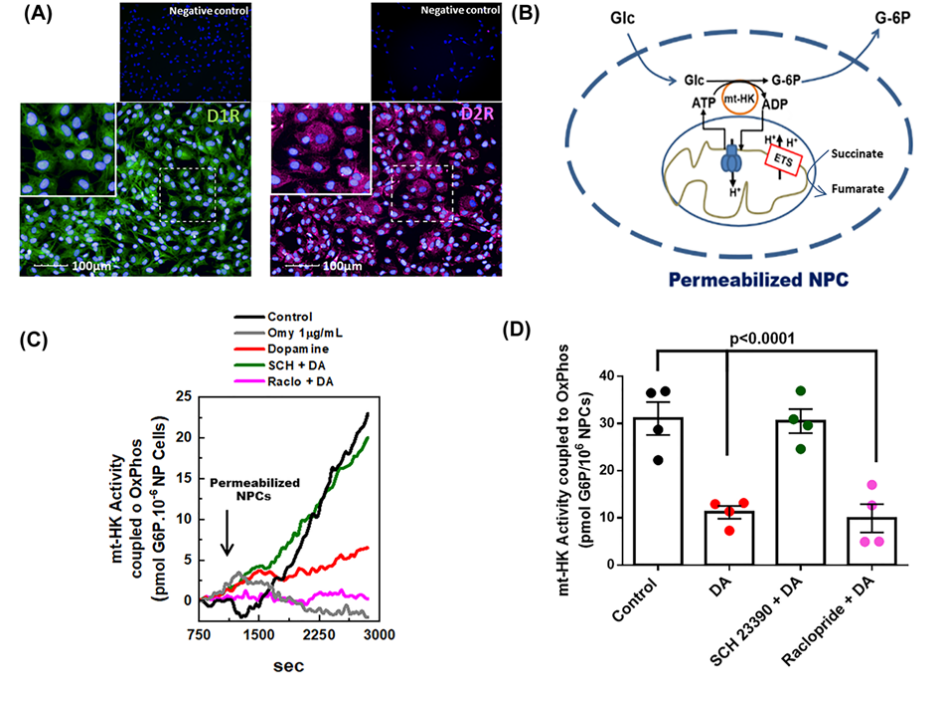
The decrease in hexokinase-coupled activity in response to dopamine is selectively blocked by D1 receptor antagonist (**A**) Representative images of NPCs labeled for D1R (green), D2R (magenta) show that NPCs express both classes of dopamine receptors. On the upper panel, representative images of the negative controls. Nuclei are stained in blue by DAPI; scale bar: 100 µm. (**B**) Scheme of the protocol used to assess the coupled-activity of mitochondrial-hexokinase (mt-HK). Addition of Succinate+ glucose + ADP + Pi couples HK activity to OxPhos. (**C**) Representative experiment of mt-HK coupled-activity in NPCs treated with control (black), oligomycin (gray) or dopamine (red), 10 µM SCH 23390 + dopamine (green) and 20 µM Raclopride + dopamine (magenta) for 48 h. (**D**) Quantification of mt-HK coupled-activity. The difference between groups was analyzed by ANOVA + Tukey’s multiple comparison test (*n*=4); D1R, dopamine receptor 1; D2R, dopamine receptor 2; ETS, electron transfer system; Glc, Glucose; G-6P, glucose-6 phosphate.

Crosstalk between dopamine and HK activity in NPCs was investigated by the analysis of classes of dopamine receptors important for the regulation of mt-HK activity. Initially, we demonstrated that human NPCs express both dopamine receptors type 1 (D_1_R) and 2 (D_2_R) (Figure 4A). D_1_R and D_2_R were blocked with specific antagonists (SCH 23390 and raclopride, respectively), and mitochondrial HK coupled activity was selectively analyzed by generation of G6P in a system stimulated by succinate + ADP + Pi, in which mitochondrial ATP is used as a substrate for mt-HK phosphorylation of glucose (Figure 4B) [43]. Control treatment with oligomycin, a specific inhibitor of F_1_F_O_-ATP synthase, completely abrogated mt-HK-dependent generation of G6P (Figure 4C). In addition to total HK activity, dopamine treatment induced a 60% decrease in mt-HK coupled activity (Figure 4C,D). The inhibition of mt-HK coupled activity was blocked by pretreatment with a D_1_R antagonist (SCH23390), but not with D_2_R antagonist (raclopride) (Figure 4C,D), demonstrating that dopamine specifically impairs mt-HK through the activation of D_1_R.

### Dopamine treatment abolishes the increase in mitochondrial calcium uptake by mt-HK

**Figure 5:**
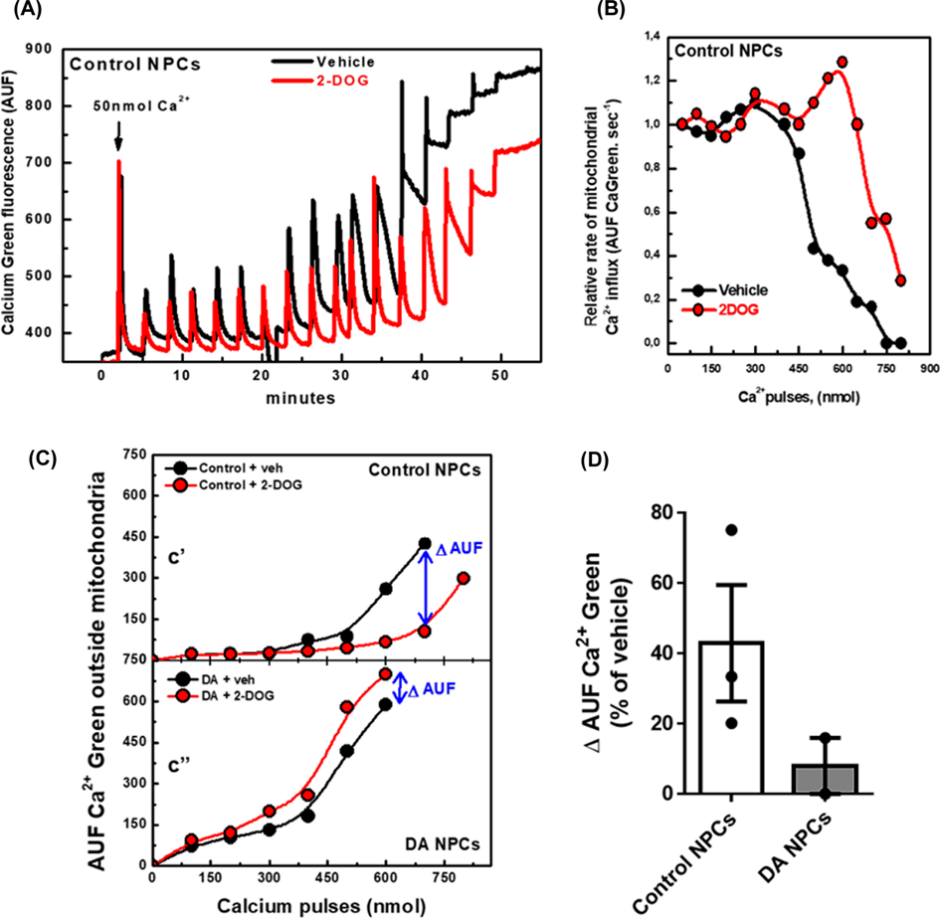
Dopamine treatment impairs the modulation of mitochondrial calcium uptake by mt-HK in NPCs (**A**) Uptake of sequential calcium pulses (50 nmol each) by mitochondria of control NPCs in the presence of 2-DOG 20 mM (red) or vehicle (black). (**B**) Rate of mitochondrial calcium influx of control NPCs in the presence of 2-DOG 20 mM (red) or vehicle (black). (**C**') Calcium accumulation outside mitochondria in response to sequential 100 nmol calcium pulses in control NPCs with 2-DOG 20 mM (red) or vehicle (black). 2-DOG shifted the curve toward calcium retention. (**C**'') Calcium accumulation outside mitochondria in response to sequential 100 nmol calcium pulses in dopamine-treated NPCs in the presence of 2-DOG 20 mM (red) or vehicle (black). Δ AUF means the difference in the calcium outside mitochondria between NPCs treated and untreated with 2-DOG. In C, each point expresses the lowest Calcium Green fluorescence after each addition of calcium, which represents the moment immediately after the uptake of calcium by mitochondria. (**D**) Quantification of Δ AUF in control (*n*=3) and dopamine-treated NPCs (*n*=2), as % of vehicle group; AUF, arbitrary units of fluorescence; Veh, vehicle.

Mitochondria play an essential role in calcium homeostasis through the uptake of cytosolic calcium. Recently, de-Souza-Ferreira (2019) demonstrated that mt-HK plays an important role in calcium uptake by brain mitochondria because mt-HK inhibition by G6P impairs mitochondrial uptake of calcium, whereas mt-HK activation by 2-DOG improves the uptake [29]. Treatment with dopamine decreased mt-HK coupled activity (Figure 4C,D); hence, we investigated whether the uptake of calcium by the mitochondria is changed in NPCs exposed to dopamine. We assessed the uptake in permeabilized NPCs using Calcium Green-5N fluorescence [43]. In control NPCs, activation of mt-HK by 2-DOG enhanced calcium retention capacity of mitochondria measured as Calcium Green fluorescence after calcium pulses (Figure 5A). The amount of calcium retained by the mitochondria represents a balance between the influx and efflux rates. The influx rate was derived from the decay of the signal of Calcium Green fluorescence. On the other hand, the accumulation of calcium in the medium results from inhibition of the influx and/or an increase in the efflux of calcium by mitochondria. The rate of calcium influx in control NPCs was gradually inhibited by calcium added to the reaction (Figure 5B, black circles); this effect was detected only at higher concentrations of calcium in NPCs exposed to 2-DOG (Figure 5B, red circles). A reduction in the accumulation of calcium outside of mitochondria upon stimulation with 2-DOG compared with that in unstimulated control NPCs indicated more efficient calcium uptake in the presence of 2-DOG (Figure 5C'). Conversely, after treatment with dopamine, the stimulation of mt-HK activity did not improve the uptake of calcium by the mitochondria because 2-DOG did not alter calcium accumulation outside of mitochondria (Figure 5C''). These data are summarized as the magnitude of the effect of 2-DOG on the decrease in the amount of calcium outside of mitochondria in control and dopamine-treated NPCs (Figure 5D).

Overall, these data demonstrated that dopaminergic signaling in human neural progenitor cells affects the redox function of mitochondria-bound hexokinase to eventually impair normal modulation of mitochondrial calcium handling.

**Figure 6:**
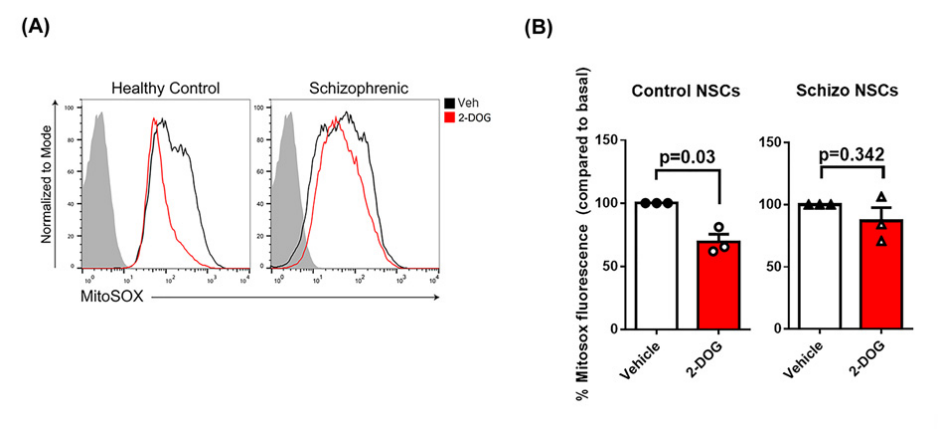
In NSCs derived from iPSCs of schizophrenic patients mt-HK is unable to modulate mitochondrial ROS (**A**) Representative experiments of flow cytometry for MitoSOX Red fluorescence with (red) or without (black) 2-DOG 35 mM in NSCs derived from healthy individuals or schizophrenic patients-iPSCs. The gray area corresponds to the negative control (without MitoSOX Red). (**B**) Quantification of MitoSOX Red fluorescence in response to 2-DOG 35 mM by control and schizophrenic-derived NSCs (*n* = 3 per group). The basal ROS was plotted as 100% and the fluorescence of MitoSOX Red after 2-DOG was normalized by the basal ROS (without 2-DOG). The differences between groups were analyzed by unpaired *t*-test with Welches correction in b (*n* = 3 per group); Veh, vehicle.

### Mitochondrial hexokinase in NSCs derived from Schizophrenia patients is unable to decrease mitochondrial ROS

Dysregulation of dopamine signaling was proposed to be involved in the pathophysiology of psychiatric disorders, such as schizophrenia [6–8,44]; hence, we compared the function of mt-HK in neural stem cells derived from iPSCs of three schizophrenia patients to that in NSCs from three healthy individuals (Table 1). We did not detect any apparent functional alterations in the oxygen flow and mt-HK coupled activity (Supplementary Figure S1). However, mt-HK in NSCs derived from schizophrenia patients was unable to decrease the generation of ROS by mitochondria, as shown in a representative experiment using MitoSOX fluorescence (Figure 6A) and from data obtained when NSCs derived from three healthy and three schizophrenia patients were treated with 2-DOG to activate mt-HK (Figure 6B). These data suggest that modulation of mitochondrial ROS by mt-HK is altered in schizophrenia.

**Table 1:** iPSC lines used for NSCs generation

Cell identification	Group	Gender	Age	Cells source	Diagnosis
CF1	Control	M	37	Fibroblast	Healthy
CF2	Control	M	31	Fibroblast	Healthy
GM23279A	Control	F	36	Fibroblast	Healthy
EZQ4	Schizophrenia	M	42	Fibroblast	Paranoid Schizophrenia
GM23760B	Schizophrenia	M	26	Fibroblast	Paranoid Schizophrenia
GM23761B	Schizophrenia	F	27	Fibroblast	Schizoaffective Disorder

## Discussion

The present study showed that dopamine impairs the modulation of mitochondrial ROS by decreasing mt-HK activity via D_1_R-dependent signaling in human NPCs. This effect resulted in an increase in extracellular H_2_O_2_ and impairment of mitochondrial calcium dynamics in dopamine-treated NPCs.

Initially, we determined whether dopamine treatment influences ROS production in human NPCs since ROS are naturally produced during enzymatic metabolism of dopamine or nonenzymatic autoxidation of dopamine degradation products [45]. Surprisingly, intracellular ROS levels were not altered by dopamine treatment (Figure 1A) in contrast with the results of previous studies that suggest that cytosolic dopamine may induce oxidative stress in brain cells [40,41]. However, all previous studies investigated dopamine metabolism in adult brain cells and not in neural progenitor cells. Products of dopamine autoxidation have been implicated in oxidative stress, leading to mitochondrial dysfunction [45]. Since dopamine treatment did not alter intracellular ROS levels in NPCs, mitochondrial function was expected to be unaffected in these cells, which was confirmed by high-resolution respirometry (Figure 2C). Normal mitochondrial function in NPCs upon dopamine treatment can be a consequence of immature state of mitochondria in progenitor cells characterized by different vulnerability to challenges [29] or may suggest that dopamine effects were not due to autoxidation and were mediated by the activation of a cell signaling cascade.

Although the levels of intracellular ROS remained unaltered, the release of H_2_O_2_ in response to dopamine treatment of human NPCs was significantly higher (Figure 1B,C). H_2_O_2_ acts as an important mediator in the extracellular medium by modulating redox-sensitive molecules and signalling pathways. Previous studies demonstrated a relevant role of H_2_O_2_ in the modulation of cellular function; for example, H_2_O_2_ inhibited dopamine release in striatal slices, which may directly impact brain function [17,46]. Accordingly, an increase in H_2_O_2_ release can down-regulate the levels of dopamine and consequently protect NPCs against dopamine neurotoxicity.

mt-HK has been shown to be a potent modulator of mitochondrial ROS in neurons, and activation of mt-HK almost completely abolishes ROS production [25]; hence, we evaluated this effect in NPCs. In control NPCs, activation of mt-HK with 2-DOG down-regulated ROS levels in intracellular (Figure 2A) and extracellular media (Figure 2B). However, dopamine treatment impaired the regulation of ROS by mt-HK since the activation of mt-HK in treated NPCs did not change the levels of ROS (Figure 2A and B). In agreement with this result, the total HK activity of dopamine-treated NPCs was significantly lower than that in the control cells (Figure 3A), which may explain the loss of ROS modulation by mt-HK after exposure to dopamine. To the best of our knowledge, there are no previous studies that correlated dopamine with mitochondrial hexokinase activity or its modulation of ROS. On the other hand, an alteration in dopamine homeostasis in human neuroblastoma cells was shown to lead to a significant reduction in the expression of VDAC [47], which anchors mt-HK to the OMM and plays an important role in mt-HK function and coupling to OxPhos. Indeed, *de novo* mutations on VDAC or alterations in specific proteins involved in the binding of hexokinase to mitochondria may impact ROS modulation by mt-HK [48,49]. The effect of dopamine on NPCs was apparently specifically related to mt-HK function because of a lack of detectable alterations in mitochondrial function (Figure 2C).

Alterations in brain glucose metabolism and in the function and protein expression of HK have been previously associated with psychiatric disorders [50,51]. Although only a few studies used patient-derived neural cells, a decrease in the attachment of HK to mitochondria in the postmortem cortex of patients with schizophrenia, bipolar disorder and depression has been demonstrated [52]. Importantly, a decrease in the activity of HK in the schizophrenic prefrontal cortex has also been demonstrated [53].

Specific antagonists of D_1_R (SCH23390) or D_2_R (raclopride) were used to investigate the mechanism by which dopamine affects mt-HK in human NPCs. We demonstrated that NPCs used in the present study expressed both dopamine receptors (Figure 4A) in agreement with the data of previous studies that described the expression of DRs in neural progenitors [54,55]. Selective assay of mt-HK activity associated with OxPhos [43] demonstrated that mt-HK activity in dopamine-treated NPCs was 60% lower than that in control NPCs (Figure 4C,D). Additionally, blockade of D_1_R signaling, but not D_2_R signaling, completely abrogated dopamine inhibition of mt-HK coupled activity (Figure 4C,D). Therefore, our data indicated that mt-HK is a target of the effect of dopamine via the D_1_R signaling pathway. In contrast, although HK activity was not evaluated in other studies, D_2_R signaling, but not D_1_R signaling, was shown to modulate glucose metabolism in the brain [56,57]. In fact, a study suggested that both D_1_R and D_2_R may regulate extracellular lactate and glucose concentrations in the brain; however, the study used apomorphine, a mixed D_1_R/D_2_R activator, which does not discriminate between receptors responsible for the effect [58]. On the other hand, during neurodevelopment, D_1_R is more abundant than D_2_R in the striatum and frontal cortex, and previous studies suggested that the effects of D_1_R overcome D_2_R signaling in the control of the proliferation of neural progenitors *in vivo* [3,59] in agreement with our data on dopamine signaling via D_1_R in human NPCs.

Recently, mt-HK was characterized as a potent modulator of calcium handling in brain mitochondria due to an increase in calcium uptake upon activation of mt-HK [29]. The present study is the first to show that mt-HK activation by 2-DOG in human NPCs also improved mitochondrial calcium uptake (Figure 5). Moreover, dopamine completely abolished the stimulatory effect of mt-HK on calcium handling since mt-HK activation in dopamine-treated NPCs did not shift the curve toward calcium retention (Figure 5C,D). mt-HK together with adenylate translocator (ANT) and outer membrane porin was proposed to be a part of a complex that may include the permeability transition pore [60]; hence, an impairment in mt-HK activity (Figure 4C) induced by dopamine may explain the impact on the modulation of calcium handling by mt-HK in dopamine-treated NPCs. Moreover, our data are in agreement with previously reported alterations in calcium handling in dopamine-treated neuroblastoma cells and a substantial decrease in the uptake of calcium by mitochondria after exposure to dopamine [46].

Finally, we evaluated the function of mitochondria and mt-HK in neural stem cells derived from three schizophrenia patients. We did not detect any significant differences in mitochondrial oxygen flux (Supplementary Figure S1a) between NSCs derived from healthy and schizophrenic subjects. Other studies used patient-derived cells and reported impaired mitochondrial respiration in iPSCs [61] and during neuronal differentiation [62]. However, another study showed that mitochondrial function in iPSCs derived from schizophrenia patients was similar to that in healthy individuals [63]. Investigation of mt-HK function indicated that OxPhos-coupled mt-HK activity was similar in the groups (Supplementary Figure S1b); however, the modulation of mitochondrial ROS by mt-HK was impaired in NSCs derived from schizophrenia patients. In NSCs derived from healthy individuals, the activation of mt-HK by 2-DOG significantly decreased mitochondrial ROS production (Figure 6A,B). However, this modulation was not detected in NSCs derived from schizophrenia patients (Figure 6A,B). mt-HK activity was measured in permeabilized cells; thus, intracellular microenvironment was lost [43], which could have masked functional differences potentially detectable in a more physiological system. Therefore, we used flow cytometry of intact cells to demonstrate that the modulation of mitochondrial ROS production by mt-HK is absent in NSCs derived from schizophrenia patients (Figure 6).

Most data on dopaminergic alterations in schizophrenia involve signaling via D_2_R. However, D_2_R signaling alterations are associated with schizophrenia symptomatology in adults, as medication used for controlling positive symptoms are mostly D_2_R antagonists [64,65]. In the present study, we used neural progenitor cells (NPCs) and neural stem cells (NSCs), which recapitulate very early steps of neurodevelopment [33] that involves a completely different set of signaling cascades and modulations. Recently, D_1_R activity was demonstrated to increase the formation of cerebral organoid by human NSCs through modulation of proliferation and differentiation [66]. On the other hand, most NSCs do not express D_2_R [67].

Overall, our data highlight the role of mitochondrial hexokinase, a potent redox modulator in mature neurons, as an equally relevant redox modulator in human neural progenitor cells. Importantly, we propose that mt-HK is a novel target of D_1_R-mediated dopaminergic signalling, which impairs the role of mt-HK as a redox modulator in NPCs. Similarly, impaired control of ROS production by mt-HK was shown in NSCs derived from schizophrenia patients; these findings may indicate a correlation between dysregulation of dopaminergic signalling during neurodevelopment with pathophysiology of schizophrenia. Applicability of these findings to other cell types in addition to neural progenitor cells used in the present study remains to be determined. The results of the present study obtained using various approaches suggest that redox balance fine-tuned by mt-HK may be disrupted by dopamine signalling in human brain cells.

## Supplementary Material

Supplementary Figure S1

## Data Availability

The data that support the findings of this study are openly available in Mendeley Data at http://dx.doi.org/10.17632/hxk7pzfk26.2.

## Abbreviations

β-NAD: β-nicotinamide adenine dinucleotide
2-DOG: 2-deoxyglucose
AA: Antimycin A
AmR: Amplex Red
Ap5A: P1,P5-Di(adenosine-5')pentaphosphate
ATP: adenosine 5′-triphosphate
cAMP: cyclic AMP
CNS: central nervous system
CRC: calcium retention capacity
D_1-2_R: dopamine D1 or D2 receptor
DA: dopamine
DAPI: 4′,6′-diamino-2-phenylindole
DHE: dihydroethidium
DMEM: Dulbecco’s Modified Eagle’s Medium
EDTA: ethylenediamine tetraacetic acid
EGTA: egtazic acid
ESC: embryonic stem cell
ETS: electron transfer system
FCCP: carbonyl cyanide-p-trifluoromethoxyphenylhydrazone
G6P: glucose-6-phosphate
G6PDH: glucose-6-phosphate dehydrogenase
Glc: glucose
H_2_O_2_: hydrogen peroxide
HK: hexokinase
IMM: internal mitochondrial membrane
iPSC: induced-pluripotent stem cell
mt-HK: mitochondrial hexokinase
NaF: sodium fluoride
NPC: neural progenitor cell
NSC: neural stem cell
OMM: outer mitochondria membrane
Omy: oligomycin
OxPhos: oxidative phosphorylation
O^•−^: superoxide anion
ROS: reactive oxygen species
SEM: standard error of mean
SOD: superoxide dismutase
VDAC: voltage-dependent anion channel
