# Supplementary material for: Dopamine signaling impairs ROS modulation by mitochondrial hexokinase in human neural progenitor cells
Source: Biosci Rep. 2021 Dec 8;41(12):BSR20211191. doi: 10.1042/BSR20211191 (PMC8661505; doi:10.1042/BSR20211191)
Supplement: Supplementary Figure S1 [file BSR-2021-1191_supp.pdf]

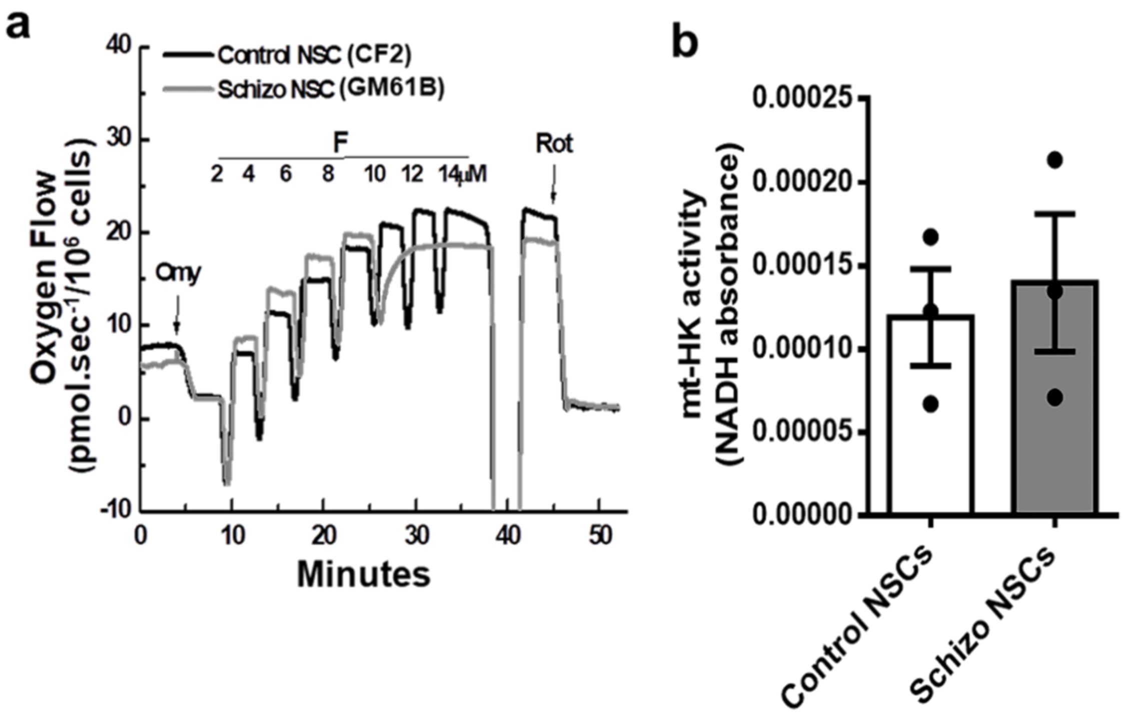

Supplementary Figure 1: NSCs derived from schizophrenic and control individuals present similar mitochondrial function and mt-HK coupled-activity. (a) Representative data of mitochondrial function of control and schizophrenia patients-derived NSCs in response to sequential additions of 1 µg/mL Oligomycin (Omy), 1 µM pulses of FCCP and 2,5 µM Antimycin A (Ama). (b) Quantification of mt-HK activity measurement from control and schizophrenic-derived NSCs. The difference between groups was analyzed by unpaired t-test (n=3 per group).
